# Lineage-Specific Biology Revealed by a Finished Genome Assembly of the Mouse

**DOI:** 10.1371/journal.pbio.1000112

**Published:** 2009-05-26

**Authors:** Deanna M. Church, Leo Goodstadt, LaDeana W. Hillier, Michael C. Zody, Steve Goldstein, Xinwe She, Carol J. Bult, Richa Agarwala, Joshua L. Cherry, Michael DiCuccio, Wratko Hlavina, Yuri Kapustin, Peter Meric, Donna Maglott, Zoë Birtle, Ana C. Marques, Tina Graves, Shiguo Zhou, Brian Teague, Konstantinos Potamousis, Christopher Churas, Michael Place, Jill Herschleb, Ron Runnheim, Daniel Forrest, James Amos-Landgraf, David C. Schwartz, Ze Cheng, Kerstin Lindblad-Toh, Evan E. Eichler, Chris P. Ponting, The Mouse Genome Sequencing Consortium

**Affiliations:** 1 National Center for Biotechnology Information, Bethesda, Maryland, United States of America; 2 MRC Functional Genomics Unit, Department of Physiology, Anatomy and Genetics, University of Oxford, Oxford, United Kingdom; 3 The Genome Center at Washington University, St. Louis, Missouri, United States of America; 4 The Broad Institute of MIT and Harvard, Cambridge, Massachusetts, United States of America; 5 Department of Medical Biochemistry and Microbiology, Uppsala University, Uppsala, Sweden; 6 Laboratory for Molecular and Computational Genomics, University of Wisconsin-Madison, Madison, Wisconsin, United States of America; 7 Department of Genome Sciences and Howard Hughes Medical Institute, University of Washington, Seattle, Washington, United States of America; 8 The Jackson Laboratory, Bar Harbor, Maine, United States of America; 9 Waisman Center, University of Wisconsin-Madison, Madison, Wisconsin, United States of America; 10 McArdle Laboratory for Cancer Research, University of Wisconsin School of Medicine and Public Health, Madison, Wisconsin, United States of America; New England Biolabs, United States of America

## Abstract

The mouse (*Mus musculus*) is the premier animal model for understanding human disease and development. Here we show that a comprehensive understanding of mouse biology is only possible with the availability of a finished, high-quality genome assembly. The finished clone-based assembly of the mouse strain C57BL/6J reported here has over 175,000 fewer gaps and over 139 Mb more of novel sequence, compared with the earlier MGSCv3 draft genome assembly. In a comprehensive analysis of this revised genome sequence, we are now able to define 20,210 protein-coding genes, over a thousand more than predicted in the human genome (19,042 genes). In addition, we identified 439 long, non–protein-coding RNAs with evidence for transcribed orthologs in human. We analyzed the complex and repetitive landscape of 267 Mb of sequence that was missing or misassembled in the previously published assembly, and we provide insights into the reasons for its resistance to sequencing and assembly by whole-genome shotgun approaches. Duplicated regions within newly assembled sequence tend to be of more recent ancestry than duplicates in the published draft, correcting our initial understanding of recent evolution on the mouse lineage. These duplicates appear to be largely composed of sequence regions containing transposable elements and duplicated protein-coding genes; of these, some may be fixed in the mouse population, but at least 40% of segmentally duplicated sequences are copy number variable even among laboratory mouse strains. Mouse lineage-specific regions contain 3,767 genes drawn mainly from rapidly-changing gene families associated with reproductive functions. The finished mouse genome assembly, therefore, greatly improves our understanding of rodent-specific biology and allows the delineation of ancestral biological functions that are shared with human from derived functions that are not.

## Abstract 2

A finished clone-based assembly of the mouse genome reveals extensive recent sequence duplication during recent evolution and rodent-specific expansion of certain gene families. Newly assembled duplications contain protein-coding genes that are mostly involved in reproductive function.

## Author Summary

The availability of an accurate genome sequence provides the bedrock upon which modern biomedical research is based. Here we describe a high-quality assembly, Build 36, of the mouse genome. This assembly was put together by aligning overlapping individual clones representing parts of the genome, and it provides a more complete picture than previous assemblies, because it adds much rodent-specific sequence that was previously unavailable. The addition of these sequences provides insight into both the genomic architecture and the gene complement of the mouse. In particular, it highlights recent gene duplications and the expansion of certain gene families during rodent evolution. An improved understanding of the mouse genome and thus mouse biology will enhance the utility of the mouse as a model for human disease.

## Introduction

The mouse (*Mus musculus*) occupies a singular position in genetics and genomics. It is both the premier animal model for human disease and development and the mammalian genome against which human DNA, genes, and genomes are most frequently compared. Despite approximately 90 million years of independent evolution [1], the laboratory mouse remains an excellent model for many human phenotypes and thus is critical to the study of human disease and mammalian development. Its small size and rapid breeding cycle are of immense practical utility, and the mature mouse genetics system, with hundreds of inbred lines, allows phenotypic consequences of sequence variation to be inferred [2].

Given the critical role of the mouse as a model organism, it is particularly important to separate shared ancestral characteristics that have been conserved in the mouse and human since their divergence from derived characteristics that are unique to either lineage. Mouse and human genes whose coding sequences have scarcely changed since the last common ancestor and that have remained unduplicated in each lineage are the most likely to have retained their ancestral functions. In contrast, genes that have duplicated along the rodent lineage may contribute to derived traits that are less relevant to human biology, and are thus less appropriate models of human physiology and disease.

Genomic duplication and divergence is a primary source of functional innovation [3]. Recent gene duplicates are highly sequence-similar and are expected to be embedded within segmentally duplicated regions of the genome (defined as DNA segments greater than 1 kb showing >90% sequence identity [4]). Approximately 40% (50 Mb) of segmentally duplicated sequences are known to be copy number variable among laboratory strains [5], whereas the remainder appears to have been fixed, either by genetic drift or by positive selection when the duplicated genes provide a selective advantage [6],[7]. Past episodes of positive selection upon codon substitutions can be inferred from evolutionary analyses of segmentally duplicated and lineage-specific genes [8]–[10].

**Figure 1:**
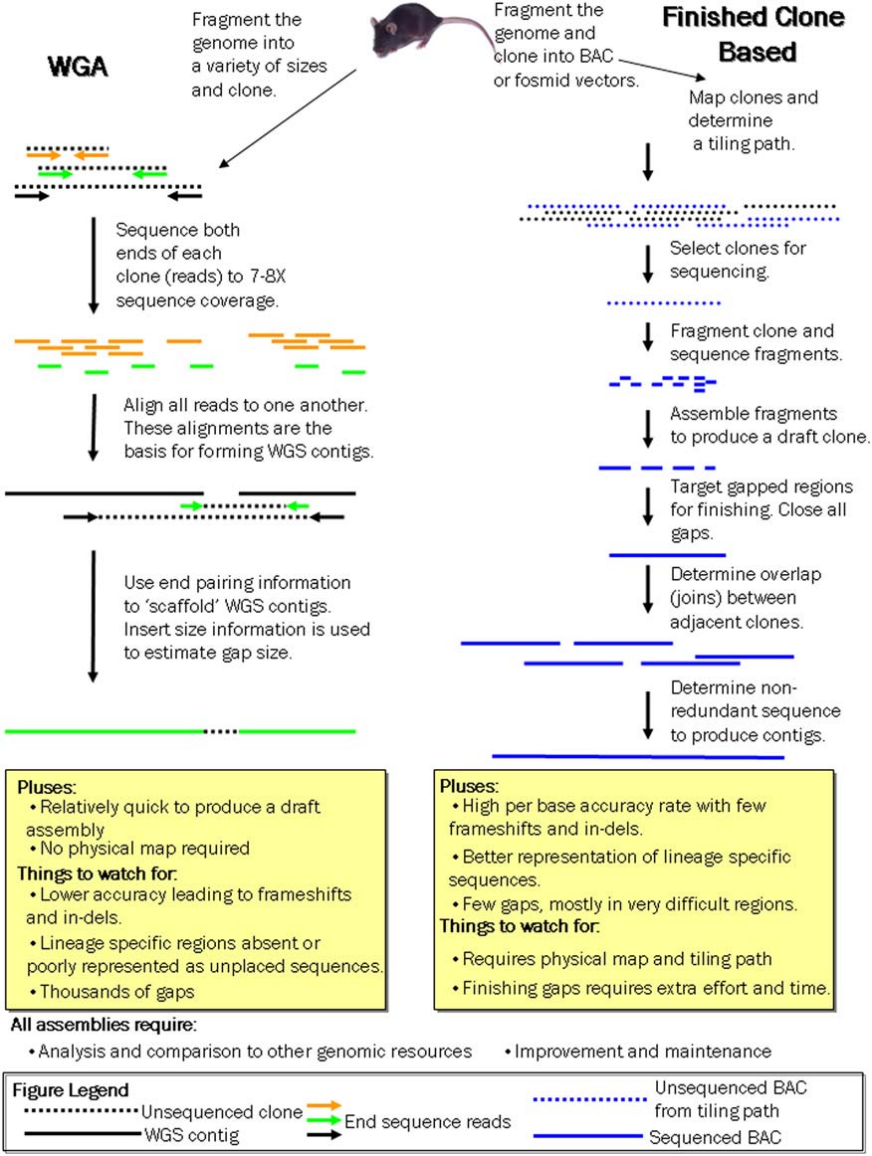
Graphical representation of the two sequencing strategies used for mouse. A careful cost/benefit analysis must be performed when approaching a genomic sequencing project. If lineage-specific biology is important, clone-based finishing of some form will be required.

In late 2002, we published a draft mouse genome assembly, referred to as the MGSCv3, of a single, inbred strain (C57BL/6J, or “B6”) [11]. This publication marked a watershed in mammalian genetics and genomics, as it allowed the first genomic comparisons between mouse and human. It also represented the first publicly funded mammalian genome project using Whole Genome Sequence and Assembly (WGSA). This approach, or variations of it, has since been the primary method for producing most mammalian genomes [12]–[16] **(** Figure 1 **)**.

Despite the great utility of the initial MGSCv3 assembly, the draft genome contained over 176,000 gaps and included entire regions whose positions and/or orientations in the assembly now appear to have been in error. The most serious issue to the use of the MGSCv3 is its almost complete lack of highly sequence-similar and recently segmentally duplicated regions [17],[18]. It was our principal concern that missing segmental duplication-rich regions of the draft assembly might harbour large numbers of rapidly evolving genes whose identification might illuminate mouse-specific biology. Only by accounting for these missing genes could we obtain a comprehensive understanding of the biology that distinguishes these two species. Given this, it has been a goal of the international consortium to produce a mouse genome assembly of coverage and quality comparable to that of the human genome described in 2004 [19]. Although it was anticipated that the effort and expense required for producing such an assembly via a clone-based approach would be substantial, this would be justified by the increased fidelity, and thus utility, of the higher quality genome assembly to the biomedical research community.

Here we report the completion of this effort and present a high-quality, largely finished clone-based genome assembly of the C57BL/6J strain of mouse, here referred to as Build 36. This new assembly includes 267 Mb of sequence (Protocol S1) that was either missing or misassembled, much of which consists of repetitive and segmentally duplicated sequence (4.94% of the genome). Over 175,000 gaps in MGSCv3 have been closed, and genome-wide continuity has improved accordingly, with scaffold N50 lengths (the scaffold length in which at least half of the bases of the assembly reside) increasing from 17 Mb to 40 Mb.

The availability of finished sequence for human, and now mouse, enables more-complete surveys of protein-coding genes in both species. We now estimate that mouse and human reference genomes contain 20,210 and 19,042 protein-coding genes, respectively. The number of mouse genes had been missing or substantially disrupted in the previous MGSCv3 assembly is 2,185. The majority of these arise from rodent lineage-specific duplications, often (61%) embedded within segmentally duplicated regions that were recalcitrant to WGSA. Many of these mouse-specific genes may contribute to rodent-specific functions and, with their inclusion in the assembly, are now available for further investigation.

## Results

### Build 36 Assembly

**Figure 2:**
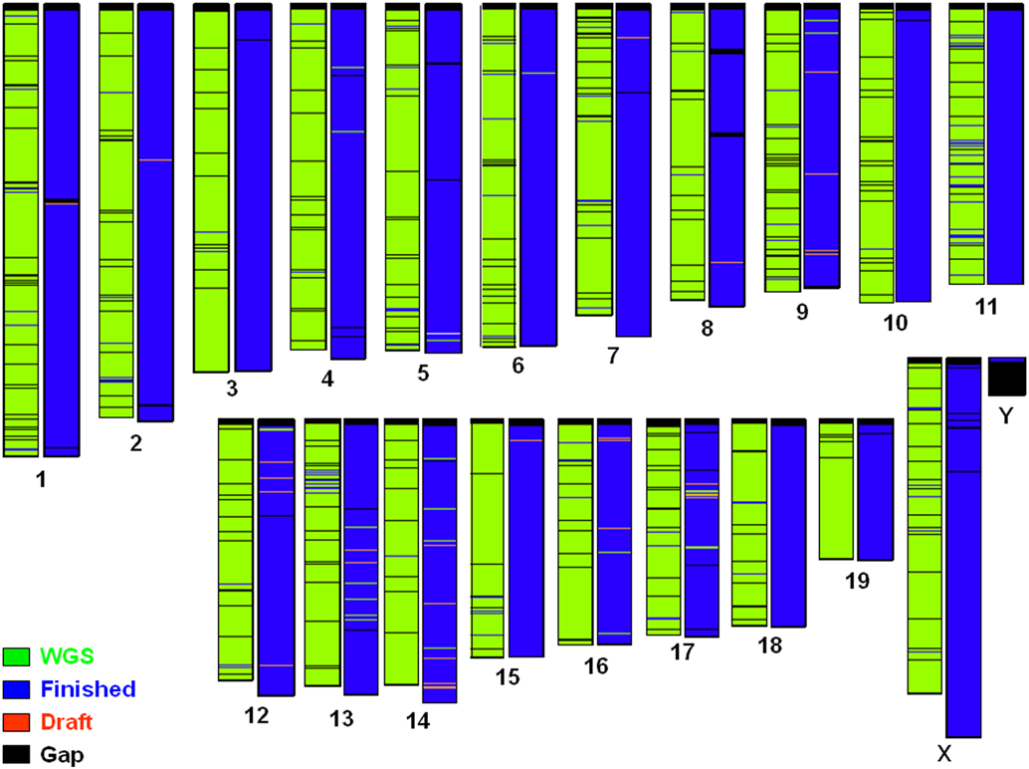
Graphical representation of sequence composition. Chromosomes are drawn to scale, with MGSCv3 to the left (green) and Build 36 to the right (purple). A female mouse provided the DNA for the MGSCv3, so no Y chromosome was available for this assembly.

**Figure 3:**
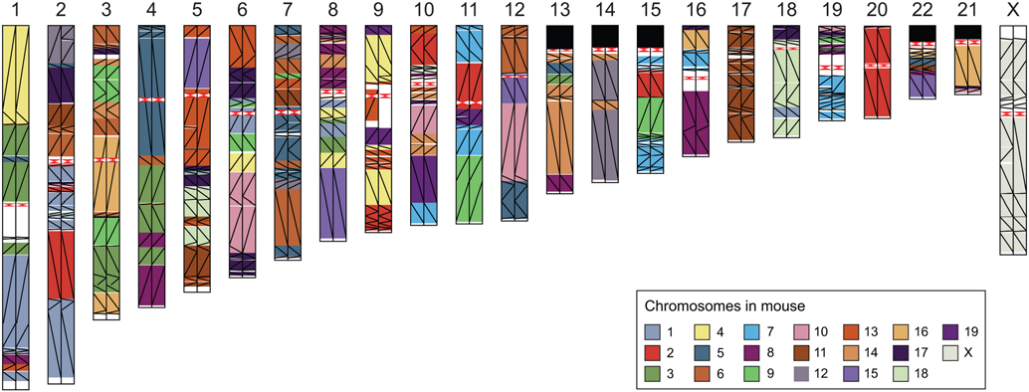
A graphical representation of conserved synteny relationships. The chromosomes of human Build 36 are painted with segments of conserved synteny ≥300 kb long with mouse MGSCv3 (left) and Build 36 (right). Colors indicate mouse chromosomes (see legend bottom right), while lines indicate orientation (top left to bottom right is direct, top right to bottom left is inverted). White regions are not covered by alignments forming a segment ≥300 kb. Red triangles are human centromeres. Note that all undirected blocks (regions of identical color) are identical between the two mouse builds except a region at the centromere of human Chromosome 9, which is itself an artifact in the MGSCv3 map. However, several areas of orientation change, some quite small, can be seen.

The mouse genome assembly (Build 36; Box 1) shows marked improvements over the MGSCv3 (Table 1) (Tables S1, S2, S3, S4, S5 and S6 in Protocol S1), with an increased amount of ordered and oriented sequence placed on a chromosome (2.58 Gb in the MGSCv3 versus 2.64 Gb in Build 36) and increased base level accuracy due to the addition of clone-based finished sequence (Figure 2) (Table S3a–S3c in Protocol S1). Scaffold continuity, as measured by the N50, is also dramatically improved, with an N50 of 40.3 Mb in Build 36 compared to an N50 of 17.8 Mb in the MGSCv3. In addition, the number of gaps in Build 36 is reduced by over 140-fold when compared to the MGSCv3 (Table 1) (Tables S4 and S5 in Protocol S1). Evidence (Box 1) indicates that Build 36 is a high-quality assembly that covers >99% of the C57BL/6J genome (assuming a 2.66-Gb genome size; see Protocol S1). Although many of the problematic regions identified in these analyses have been corrected in a subsequent Build 37 (the current public build, see Box 1 and Protocol S1), a few regions remain under review and will be addressed in forthcoming assemblies. Improvements to the assembly are evident at a fine scale in the spanning of previous gaps and inclusion of locally duplicated sequences. However, at a larger scale, the genome structure remains basically unchanged from MGSCv3, and conserved syntenic relationships to human inferred from the two assemblies have remained essentially unaltered (Figure 3).

**Box 1. Assembly Production and Quality Assurance**

The mouse genome assembly (Build 36) was produced largely as described previously [19] but with some variations in methodology and standards (Protocol S1). The availability of a high-quality WGS assembly was essential in providing a framework for the clone-based assembly. Nevertheless, over 7% of the bases found in the finished clone sequences failed to align to the MGSCv3. Unaligned sequence contributed from approximately 4% for Chromosome 11 to 18% for the X chromosome (Protocol S1: Alignments)). Tables S1, S2, S3, S4 and S5 in Protocol S1 provide assembly statistics, stratified by chromosome, for both Build 36 and the subsequent Build 37, which was produced after the analysis performed here. While this analysis led to improvements in Build 37, the changes in this build are not expected to drastically alter the conclusions of the analysis presented in this manuscript. The main differences between Build 36 and Build 37 are the incorporation of an additional 8.3 Mb of sequence onto the assembled chromosomes and 44.7 Mb of sequence as unplaced sequence. During the course of analyzing Build 36, a number of scaffolds that were unplaced in the MGSCv3 were found to contain sequences not represented in Build 36. In all cases, these sequences contained protein-coding genes. While many of these can be associated with a chromosome, the exact order and orientation is unknown. However, because of the missing gene sequences, we thought it was important to release Build 37 with these sequences included. Work is ongoing to both place these sequences in the correct location on the chromosome and to identify clone-based sequences to represent them. Although statistics are provided for the Y chromosome, analysis of this chromosome will not be discussed here, because it remains a separate project that will be described at a later date. However, the authors have generously provided the scientific community with data prior to publication (J. Alfoldi, personal communication).

To assess the accuracy of Build 36, the genome assembly was compared to several independent sources of data including a linkage map [81], a radiation hybrid map [82], and sequences (genomic and transcript based) not used to generate the assembly [83]. In all cases, the discrepancy rate was very low, indicating that Build 36 is a high-quality and high-coverage genome assembly (Protocol S1). This project was the first to use an optical map to assess the assembly and to disambiguate problematic regions. We assembled a genome-wide SwaI restriction map of the C57BL/6J mouse genome using single-molecule ordered restriction maps obtained from the optical mapping system [84]–[86]. This optical map showed 99% concordance with the restriction endonuclease digest pattern predicted by the genome assembly. We identified 423 discordant sites which were manually evaluated; in 95 cases, the optical map was judged to be correct, in 220 cases the sequence as determined to be correct and the remaining 108 cases were ambiguous (Protocol S1). The optical map provided critical data for clone placement in several repetitive regions, such as the beta-defensin region on mouse chromosome 8 (Figure S66 in Protocol S1), as well as providing evidence for clone order in regions where there was little other information, such as some pericentromeric regions. In addition, the optical map covers roughly two-thirds of the 103 unspanned gaps in Build 36 (Protocol S1: Comparison of Optical Map to Build 36 (pdf)) and will be used in future builds to provide more accurate gap estimates.

**Table 1:** Changes from MGSCv3 to Build 36 assemblies.

Parameter	MGSCv3	Build 36
**Assembled Genome**	2.685 Gb	2.661 Gb
**Non-N genome size**	2.475 Gb	2.567 Gb
**Unplaced sequence**	103.9 Mb	17.1 Mb
**N50**	17.8 Mb	40.3 Mb
**Number of gaps**	176,507	1,218
**% Segmental Duplicated sequence**	<0.1%	0.0494
**Interspersed repeats**	1.046 Gb	1.118 Gb
**LINE1**	460.1 Mb	505.3 Mb
**Number of Gene Models**	22,011^a^	20,210
**Number of Unplaced Gene Models**	n/a	191
**Number of identified 1∶1 orthologs with human genes**	12,845^a^	15,187
**% coding**	1.25%^a^	1.27%
**% utr**	0.48%^a^	0.87%

a Values for MGSCv3 protein-coding genes are taken from the gene catalogue used in the draft mouse genome publication [3].

We identified a total of 334 chromosomal breakpoint intervals between human and mouse and refined the breakpoints to an average interval length of 335 kbp. We found that 50% (167/334) of the breakpoints and that 28.7% by base pair (32.2/111.9 Mbp) intersected with segmental duplications. This 6-fold enrichment is significant (*p*<0.0001) by simulation (*n* = 10,000 replicates). Using data from rat, mouse, and human, we further categorized the breakpoint intervals as mouse-specific (*n* = 18), contiguous with rat (*n* = 276), or ambiguous (*n* = 40). The latter category frequently shared only one of the two breakpoints between mouse and rat, suggestive of breakpoint reuse [20]. While all three categories are significantly enriched in segmental duplications, we found the majority of mouse-specific breakpoints (16/18 or 89%) and ambiguous rat–mouse (37/40 or 93%) breakpoints harbored segmental duplications. These data strongly support the now longstanding observation that chromosomal rearrangements preferentially associate with regions enriched with duplicated sequences [18],[21]–[23].

### Newly Assembled Genomic DNA Consists Mostly of Lineage-Specific Sequence

The revised Build 36 assembly contains 139 Mb of sequence that could not be aligned against, and thus appears to have been absent from, the previous MGSCv3 draft assembly. 108 Mb (77%) of this sequence consists of 119,000 repetitive elements (Table S7 in Protocol S1); this was expected because highly sequence-similar repetitive sequences are particularly difficult to resolve using WGSA [24],[25]. One-third (45.2 Mb; 33%) of this newly incorporated repetitive sequence is derived from the most abundant mouse repeat, LINE1. These newly identified LINE1 copies have, on average, a markedly lower divergence from the consensus (mean 4.5%, as reported by RepeatMasker) than those that align completely to the previous MGSCv3 sequence (mean 9.4%). Hence they tend to have been inserted more recently in the mouse lineage. Insertions of LINE1s are clearly ongoing because they are known to be responsible for 10–15% of deleterious mutations [26],[27].

**Figure 4:**
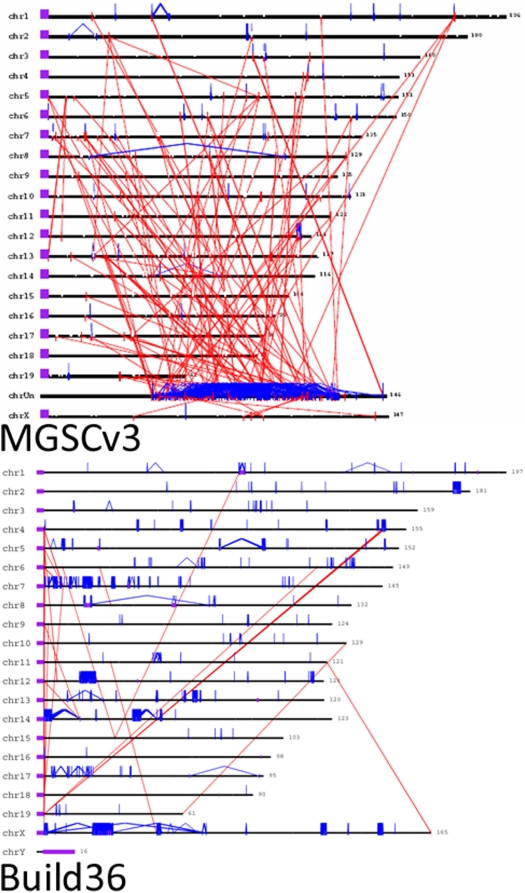
The distribution of segmental duplication in MGSCv3 (top) and Build 36 (bottom). Interchromosomal (red) and intrachromosomal (blue) duplications (>95% identity and >10 kbp) in length are shown for both genome assemblies with the requirement that pairwise alignments are shown for only those regions (Build 36) that are also confirmed by the WGS depth of coverage analysis (black vertical bars/ticks). Positions of the centromeres (acrocentric) are shown (purple) for the MGSCv3 build. Initial estimates predicted the amount of segmental duplication to be approximately 1.5–2% of the genome. Calculations performed using Build 36 suggested the amount is much higher, approximately 4.5–5%. In addition, >60% of duplicated sequences were unplaced in the MGSCv3. In Build 36, almost all are assigned to a chromosome

Eighty percent of sequence added or corrected in the mouse genome assembly consists of segmentally duplicated regions or interspersed repeats. Most of these have now been ordered and oriented on a chromosome (Figure 4 and Protocol S1). The large amount (126 Mb; 4.94%) of segmentally duplicated sequence in the mouse genome was unexpected, because the initial MGSCv3 assembly contained virtually no (<0.1%) such sequence [4], and what was there in MGSCv3 resided in a large pool of unplaced sequences. When evaluating the segmental duplication content of Build 36, we used an assembly-independent approach [17] to validate 85–91% of long (≥10 kb) and highly similar (94–99%) duplications. Nevertheless, some virtually identical (>99%) duplications remained as artefacts in the assembly, because these exhibited slightly lower rates of validation (82%), and further work will be required to resolve them. Of critical importance, with the addition of the new data in Build 36, segmental duplications in the mouse thus are now seen to occupy a similar proportion of the genome as they do in human. However, they are overwhelmingly intrachromosomal, with a high prevalence of tandem duplication, whereas human segmental duplications are often interchromosomal [17].

This is partially addressed in [5], but we elaborate further. When one compares the divergence of segmental duplications between the mouse (Build 36) and human genome assemblies (Build 36), there are some notable differences. For example, the majority of human intrachromosomal segmental duplications show high sequence identity (98.5–99.5% with a mode at 99% sequence identity). In contrast, the pairwise sequence identity distribution for mouse segmental duplications shows a much more bell-shaped distribution with a clear mode around 95%. These findings are consistent with a burst of intrachromosomal segmental duplications in the human–great ape lineage as recently discussed [28], and indicate that perhaps intrachromosomal segmental duplications have a more ancient origin in the mouse. There are, however, some important caveats. First, it is likely that high-identity duplications in the human genome assembly have been better resolved because of the larger and longer effort in its finishing—as such, we posit that we are underestimating the highest-identity segmental duplications in the mouse genome. Second, the substitution rate for rodents is significantly higher than primates, so if divergence is used as surrogate for evolutionary age, this adjustment must be taken to account. Finally, this pattern is true for C57BL6, but the pattern in wild-type mice under strong natural selection may differ significantly. Estimating differences in timing of segmental duplications is particularly tricky in the absence of comparative sequence data of more closely related rodent genomes.

### Mouse and Human Protein-Coding Gene Repertoires

The Build 36 assembly contains many genes that were absent, truncated, incomplete, or misassembled in the initial draft MGSCv3 genome sequence. As we describe below, the vast majority of these genes reside in segmentally duplicated regions. Using gene predictions for human and mouse from both NCBI [29] and Ensembl [30], we retained only those that were conserved either within or between the two species. Gene models were assessed for their reliability by: (i) comparing the exon boundaries in alignments of predicted orthologous and paralogous genes, (ii) considering whether mouse and human homologues lay within regions of conserved synteny, and (iii) automatically inspecting genes for reading frame disrupting mutations [31]. Homologues generated by retrotranspositions since the human–mouse divergence lack conserved exon boundaries and mostly lie outside of syntenic regions, and thus could be assigned as likely pseudogenes.

This process identified 20,210 high-quality protein-coding gene models in mouse and 19,042 such models in the human genome (Protocol S1, section: Protein Coding Genes and Gene Families). This number of reliable human genes is very much lower than initial reports [11],[19], yet it compares well with three more recent estimates [32]–[34]. The marked discrepancy between mouse and human gene counts results mainly from contrasting rates at which these lineages have acquired gene duplicates, as we shall discuss below. The revised proportions of protein-coding sequence in the euchromatic sequence of the mouse and human genome assemblies are now found to be 1.27% (33.5 Mb out of 2.64 Gb) and 1.06% (32.6 Mb out of 3.08 Gb), respectively, rather than the approximately 1.5% often quoted.

### 1∶1 Orthologs

Simple 1∶1 orthologs correspond to genes that have remained intact and unduplicated since the last common ancestor of mouse and human. Using a recently developed phylogenetic approach [32], we could identify 15,187 human and mouse genes in simple 1∶1 orthologous relationships, representing 75% of mouse and 80% of human genes. By comparison, the original survey of genes from the draft MGSCv3 assembly was only able to clearly identify 58% (12,845 out of 22,011) of mouse genes as having 1∶1 orthologs in the human genome [11]. Simple 1∶1 orthologs exhibited median nucleotide and amino acid identities of 85.3% and 88.2%, respectively. These mouse and human genes differed by a median of 0.58 synonymous substitutions at synonymous sites (*d*_S_), and had a median ratio of nonsynonymous to synonymous substitutions (*d*_N_/*d*_S_) of 0.095 (Table 2).

**Table 2:** Characteristics of one-to-one orthologs predicted for human and mouse genes.

Counts of 1∶1 orthologs	151878
**dN**	0.057 (0.024–0.11)
**dS**	0.58 (0.46–0.75)
**dN/dS**	0.095 (0.043–0.18)
**Amino acid sequence identity**	88.2% (79.4%–94.7%)
**cDNA sequence identity**	85.3% (80.6%–88.8%)
**Human sequence length (codons)**	443 (283–706)
**Mouse sequence length (codons)**	443 (283–706)
**Aligned sequence length (codons)**	434 (276–693)
**Pairwise alignment coverage of the longer sequence**	97.4% (99.4%–100%)

Shown are median values and, in parentheses, lower and upper quartiles.

Only eight mouse genes with 1∶1 orthologs in human were entirely absent from the initial MGSCv3 assembly (see Table S8 in Protocol S1); a further 13 single-copy gene models in mouse that have been duplicated on the human lineage were also missing from MGSCv3. Nevertheless, 825 1∶1 orthologs were substantially disrupted in MGSCv3: at least 25% of their exonic sequence was absent from or misplaced in the draft MGSCv3 assembly (see Materials and Methods and Protocol S1). The exonic sequences of another 3,439 1∶1 orthologs were also affected by missing or misassembled sequence, albeit less drastically (see Material and Methods and Protocol S1). In total, 30% of all gene models in Build 36 would have been disrupted to some extent by errors in the MGSCv3 assembly.

### Mouse (C57BL/6J)-Specific Genes

**Figure 5:**
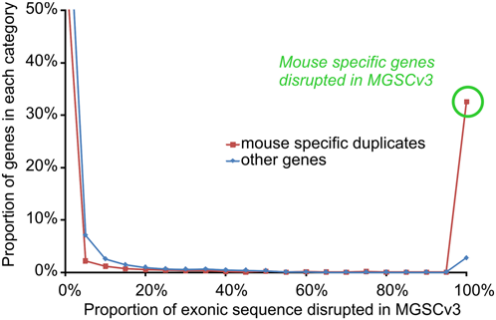
The proportion of exonic sequence disrupted in the MGSCv3. Mouse lineage-specific gene duplicates are shown in red, and all other genes are shown in blue. The large number of mouse-specific genes that are entirely missing, truncated, or otherwise disrupted in MGSCv3 underscores the value of the finished Build 36 assembly in understanding rodent-specific biology.

**Figure 6:**
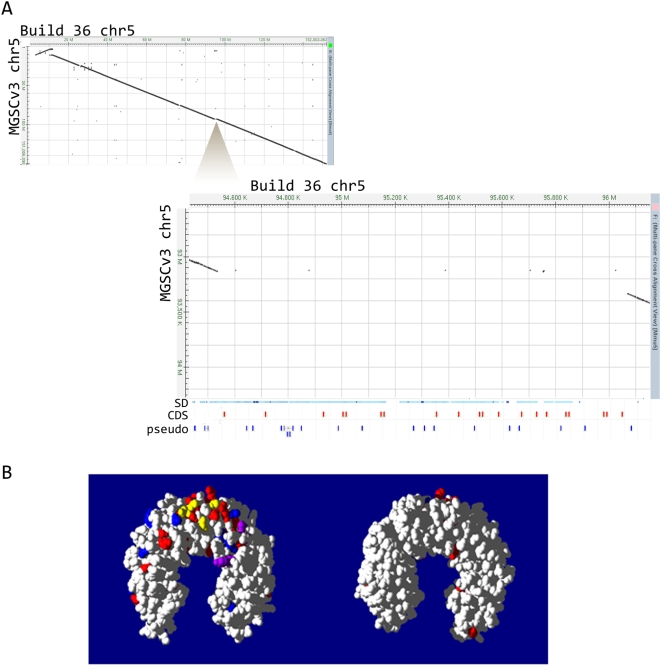
Improvement of a region in Build 36, rich in *Pramel* genes, which is virtually absent in MGSCv3. (A) The upper left hand corner shows a dot-matrix view of the Build 36 Chromosome 5 (horizontal axis) aligned to the MGSCv3 Chromosome 5 (vertical axis). The triangle marks the portion of the chromosome shown in the zoomed in view. The axes are in the same orientation. 1.5 Mb of sequence that was absent from MGSCv3 has been included in Build 36. This region contains 30 *Pramel* genes (shown in red) and approximately an additional 20 *Pramel* pseudogenes (in blue). This region consists almost entirely of segmental duplications (represented by blue lines below the dot matrix), which previously confounded the WGA algorithm. Gene models for Build 36 are displayed below the dot matrix view. SD, segmental duplication; CDS, coding regions; pseudo, pseudogenes. (B) Although the orthologous *PRAME* and *Pramel* gene families have expanded independently in the primate and rodent lineages, positive selection has been most intense on equivalent regions of their structures. Positive selection on amino acid substitution has been most intense on one exterior surface (left) and has been virtually absent from the alternate face (right). Amino acids predicted to have been positively selected among human HSA1 *PRAME* genes (shown in red), mouse MMU4 *Pramel* (blue), or rat RNO5 *Pramel* (purple) genes have been mapped onto an homologous structure (Protein Databank code 2BNH). Amino acids that are positively selected in two or more species are shown in yellow. Three positively-selected sites among mouse *Pramel* genes are not highlighted, as they occur within insertions relative to the 2BNH sequence.

It is thus clear that while MGSCv3 had provided a largely faithful representation of unduplicated 1∶1 orthologs, Build 36 provides across-the-board improvements to the quality of gene predictions. This greatly improved assembly now permits a more-complete understanding of rodent-specific genes. Of 2,185 Build 36 gene models that were substantially disrupted by missing or misassembled sequence in MGSCv3 (see Materials and Methods), 1,259 (58%) are mouse lineage-specific duplicates (Figure 5). Similarly, of 242 genes that fell entirely within gaps in the previous MGSCv3 assembly, 221 (91%) are mouse-specific duplicates; 90% of the 331 gene models that were in previously unplaced sequences in MGSCv3, but correctly assembled in Build 36, are also mouse-specific duplicates. Conversely, of the genes that were only duplicated on the human but not the mouse lineage, only 101 were disrupted in MGSCv3. The repetitive genomic regions in the mouse (an example of which can be found in Figure 6) thus presented a considerable challenge to sequencing, assembly, and gene prediction.

Of the ten gene families that have seen the greatest expansions over the mouse lineage, we find that all but two are associated with reproductive functions (Table 3). Approximately half of these genes overlap gaps in the previous MGSCv3 assembly (Figure 5). These ten families include genes with spermatid- or oocyte-specific expression, and large families of vomeronasal receptors (VRs). This suggests that conspecific competition may have driven much adaptive change in rodent gene repertoire and genome landscape.

**Table 3:** Gene families that have experienced the largest rodent-specific expansions categorized by likely function.

Gene Families	Mouse Chromosomes	Functional Category	Gene Counts		Genes Overlapping MGSCv3 Gaps	Genes Absent from MGSCv3
			Mouse	Human^a^		
***Spetex*** **/** ***Speer***	5, 14	Reproduction	111	—	42	14
**V1R**	7	Reproduction	90	—	52	23
***Pramel***	4, 5	Reproduction	90	22	55	1
**KRAB zinc finger**	2,5,7,8,10,11,12,13,16,17,19	Transcription regulation	80	5	51	9
***Slx*** **, Sycp3-like, X-linked proteins**	X	Reproduction	58	—	29	4
**IgG kappa**	12	Immunity	55	13	5	2
**V2R**	5, 7, 10, 13, 14, 17	Reproduction	47	—	20	5
***Ssty***	Y	Reproduction	55	—	55	55
**V1R**	6	Reproduction	37	—	1	0
**V1R**	13	Reproduction	35	—	3	0
**Total**	—	—	**671 (100%)**	**36**	**313 (47%)**	**113 (17%)**

a Human orthologs for many of the most rapidly expanding mouse gene families cannot be readily identified, either because of gene loss or rapid sequence divergence.

**Figure 7:**
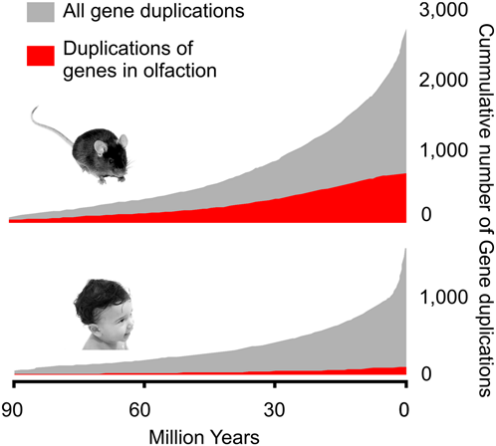
Cumulative numbers of protein-coding gene duplication events on the human and mouse lineages since their divergence (grey). Evolutionary time is estimated using *d*_S_ (the number of synonymous substitutions per synonymous site) and a divergence time of 91 million years. The greater number of genes in the mouse compared with the human genome is largely accounted for by the lower rate of olfactory and vomeronasal receptor gene duplications (red) in the primate lineage.

Gene duplicates in the rodent lineage far out-number those in the primate lineage (3,767 in mouse and 2,941 in human). In general, despite particularly fast rates of protein evolution [11], their low sequence divergence at synonymous sites implies that they have been only relatively recent additions to the mouse lineage (Figure 7). This increased number of genes in mouse compared to human is, for most families, primarily attributable to gains in the rodent lineage; however, the primate lineage has also experienced substantial losses of genes encoding olfactory receptors, VRs, VR-associated molecules, and putative pheromones [35]–[37] (Figure 7). Four of the gene families with the largest rodent-specific expansions (Table 3) encode VRs, all of which are concentrated within large segmentally duplicated regions. The concentration of VRs and predicted pheromones [38] on Chromosome 7 contributes to this chromosome's 2.7-fold enrichment in segmental duplications [5]. Approximately half of all gene duplication events that we observe in mouse appear to have occurred since the time of its divergence from the rat lineage approximately 12–14 million years ago (Mya) (Figure 7) [39].

Gene duplications have caused large expansions of gene families in primates as well, several of which were highlighted in the manuscript describing the finishing of the human genome [19]. Differences in the human and mouse gene repertoires can also arise from gene loss in the rodent lineage. Genes that are associated with Mendelian disease in human can be presumed to have critical functions, and examples of their loss in the mouse are, therefore, rare and unexpected. One such case is the human gene *EYS* (Entrez Gene (http://www.ncbi.nlm.nih.gov/gene/) GeneID 346007), whose disruption results in retinitis pigmentosa; nevertheless the mouse ortholog found in B6 is a pseudogene [40]. For the few human genes whose mouse orthologs are entirely absent from the current assembly, it is difficult to determine whether their absence reflects past deletions of genomic sequence or else indicates regions that continue to be problematic to sequence. One gene that has remained elusive since the draft MGSCv3 assembly is the mouse ortholog of human *KAL1* (GeneID 3730), whose disruption leads to an absence or hypoplasia of olfactory bulbs and tracts in human patients [41]. No transcript or genomic evidence for the existence of a *KAL1* mouse ortholog is apparent. In fact, it has been suggested that the entire genomic region surrounding the *KAL1* gene, which is adjacent to the pseudo-autosomal boundary in Xp22, in human is absent from the mouse genome [42]. However, this genomic region is not stably propagated in bacteria [43] and may, in fact, vary amongst strains [44]. Evidence of mouse orthologs for some genes in this genomic region does exist within transcript sequences that are derived from strains other than C57BL/6J [45]–[46].Completing the catalogue of mouse genes and pseudogenes and understanding their homology relationship with human genes requires additional sequence from more than a single strain.

More rarely, a mouse gene may lack an apparent human ortholog simply because rapid evolution renders any similarity in their sequences undetectable. This is the case with human *AU022751* (Mouse Genome Informatics (MGI; http://www.informatics.jax.org/) ID: 2147968; GeneID 102991) and mouse *EG624310* (MGI: 3644796 ; GeneID 624310) that lie in conserved synteny on their X chromosomes: they are orthologous, yet show only residual and nonsignificant sequence similarity in database searches. The mouse genome contains a further ten mouse paralogs of *EG624310* (MGI: 3644796 ; GeneID 624310) on a separate region of chromosome X that was largely absent from MGSCv3. *AU022751 (*MGI: 2147968; GeneID 102991) is of unknown function, but its mouse orthologs appear, from the expression profile of expressed sequence tags (ESTs), to be expressed frequently in oocytes and exhibit evidence for positive selection on amino acid substitutions (unpublished data). This demonstrates how apparently lineage-specific gene families can emerge by unusually rapid sequence divergence, driven in part by positive selection, over only tens of millions of years, and is a prominent example of the novel biology that can be discovered using a high quality mouse genome assembly.

### Mouse Gene Family Expansions and Reproduction

The largest rodent-specific expansions have occurred among sperm-associated glutamate (E)-rich (*Speer*) genes on Chromosomes 5 and 14. Most *Speer* genes appear to have roles in spermatogenesis [47],[48] but some *Speer*-homologous genes, termed α-takusans, are expressed in the mouse brain and regulate synaptic activity [49]. *Speer*/α*-takusan* genes are absent from nonrodent genomes because they arose via a partial gene duplication of *Dlg5 (*MGI: 1918478; GeneID 71228) [47], followed by multiple duplications and sequence diversification, early in the rodent lineage. Many (24) of these *Speer* homologues were absent from or disrupted in the MGSCv3 because of earlier assembly problems associated with their repetitive nature. Assembly gaps remain within Build 36 Chromosome 14, hinting at yet more undiscovered *Speer* genes.

Members of the preferentially expressed antigen of melanoma (*PRAME*) gene family made up the third largest gene family with mouse specific expansions. All 90 mouse *Prame*-like (*Pramel*) genes were the result of duplications after the divergence of the rodent and primate lineages [8], and the majority of these were also incomplete in MGSCv3 (Figure 6). In primates, this family has also seen much independent expansion, and it is highly divergent in copy number both between species and within the human population [8],[16]. In rodents, *Pramel* gene duplications occurred not only locally, in *cis*, but also by translocation of a single *Pramel* gene from the ancestral Chromosome 4 to Chromosome 5, with additional *cis*-duplications there subsequently. A further segmental duplication, absent in the rat, seeded continued expansion of this gene family within chromosome 5 (Figure S1 in Protocol S1). There is abundant evidence for past episodes of positive selection within rodent *Pramel* and primate *PRAME* genes (Figure 6) [8]. Little is known of the functions of mouse *Pramel* genes, except that they are often expressed in oocytes, in early embryos, and in spermatogonia [50].This expression profile suggests that they might perform important mitotic roles in rapidly dividing cells.

Extensive duplications within two further gene families have been restricted to X and Y chromosomes (Table 3). These families derived originally from ancestral autosomal paralogs: *Sycp3* (MGI: 109542; GeneID 20962) on Chromosome 10 [51], and *Spin1* (MGI: 109242; GeneID 20729) on Chromosome 13 [52]. *Sycp3* (MGI: 109542; GeneID 20962 ) gave rise to *Slx* (MGI: 99543; GeneID 664829) and *Sly* (MGI: 382301; GeneID 382301) genes on the X and Y chromosomes, respectively. The *Spin1* homologues on the sex chromosomes, including *EG546176* (MGI: 3645924; GeneID 546176) and *Ssty1* (MGI: 1314663; GeneID 20611), appear to have arisen through retro-transposition and subsequent duplication. Nevertheless, the maintenance of open reading frames over the long evolutionary distances separating the divergent X and Y copies suggest that protein coding potential has been maintained for many of these genes. The expression of both gene families is specific to spermatids [53],[54]. Translocation to the sex chromosomes and subsequent widespread duplications may reflect advantages when these genes are hemizygous in males [55] or when gender conflict affects the sex ratio of mouse progeny [56]. Multiple copies of these genes may act to avoid X inactivation [57] or help to compensate for lack of recombination on the Y chromosome [58].

We found that many genes in the four families described above—namely *Speer*, *Pramel*, *Slx*/*Sly*, and *EG546176*/*Ssty1*—have experienced extensive positive selection upon amino acid substitutions (unpublished data), suggesting that duplication events were themselves fixed preferentially in ancestral mouse populations. The preponderance among rodent-lineage specific genes of those with reproductive function provides an indication of the prominent role of conspecific competition in shaping the mouse gene repertoire and hence its biology [59]. Despite their large number and the important roles that they have played in the emergence of the laboratory mouse, these genes are poorly represented among current gene catalogues. This is because they are generally found as multiple tandem copies in segmentally duplicated genomic regions, accompanied by significant numbers of gene fragments and pseudogenes. Only now, with the availability of a high-quality finished mouse genome, can the crucial roles of these rapidly evolving genomic regions in determining lineage-specific biology be fully appreciated.

### Mouse Non–Protein-Coding RNA Genes

The transcribed and functional portion of the mouse genome consists of noncoding as well as protein-coding genes. Hundreds of microRNA loci, for example, have been detected within recent mouse genome assemblies [60]. In addition, thousands of long noncoding RNAs (ncRNAs) have been detected from full-length mouse cDNA libraries [61],[62]. The contribution of these long ncRNAs to mouse biology, however, remains a matter of extensive debate. Evolutionary studies have yet to contribute to this debate by distinguishing long ncRNAs that have single human orthologs from others that have duplicated, or else emerged de novo, on the mouse lineage.

Evidence for conserved transcription is apparent for only a small proportion of long mouse ncRNA sequences, in contrast to protein-coding genes. Of 3,051 well-documented mouse long ncRNA sequences [63], only half (1,538 of 3,051; 50.4%) can be mapped to the human genome assembly using cross-species genomic alignments. This proportion is slightly higher than the fraction (39%) of all mouse nucleotides that have been aligned to the human genome assembly. Of these 1,538 mapped sequences, only 439 (14% of 3,051) have EST or cDNA evidence for orthologous transcription in human. Although most mouse long ncRNAs lack evidence of human expression, the minority with conserved expression represents a statistically significant (*p*<10^−3^) 9.5-fold enrichment over what is expected from random genome sampling. These 439 ncRNAs have a median length of 1,920 bp and are significantly less likely to contain annotated repetitive sequence (13% of nucleotides overlap mouse repeats) than either random intergenic regions ( 45%, *p*-value<0.001) or their nonconserved counterparts (25%, *p*-value<0.001). These mouse ncRNAs should now be prioritized for further experimental scrutiny. The remaining mouse long ncRNAs that appear to lack human orthologs may represent rodent-specific biology, transcriptional noise, or transcribed noncoding sequence that remains, as yet, unidentified in humans.

### Mouse Strain-Specific Genomic Sequence

Rodent lineage-specific sequence includes regions that are copy number variable among laboratory mouse strains. Indeed, many of the largest rodent-specific gene families are known to be copy number variable among mouse strains [5] including *Slx*, V1R, and V2R family genes. We used read pair data to identify larger scale structural events in Build 36. Read pairs from nine non-B6 strains identified 2,573 sequence differences where B6 contains an insertion relative to the other strains, at least 263 of which appear to be at least 10 Kb in size. Notably, 604 events were identified that define potential deletions in B6 relative to other strains, including deletions to the pseudo-autosomal region of the X chromosome. These deletions would represent sequence present in the mouse population but absent from the reference genome. In contrast, using C57BL/6J read pairs as a negative control, we observed only 96 possible insertion events, 42 possible deletion events, and 67 possible inversion events (Table S9a and S9b in Protocol S1). Clearly, to move towards a comprehensive catalogue of CNVs, and indeed all variation including quiet mutations [64], in *Mus musculus*, sequencing of additional mouse strains will be required.

## Discussion

The mouse genome assembly (Build 36) is now of high fidelity and completeness, and its quality is comparable to, or perhaps better than, that of the reference human genome assembly. The finished mouse genome adds over 6% additional euchromatic sequence, much of it repetitive, but includes 1,259 mouse-specific genes that were missing or grossly misassembled in the draft. Improvements to the assembly should enhance many coordinated initiatives that are exploiting the utility of the laboratory mouse for understanding human biology and disease processes. For example, an international effort to establish baseline phenotypic measurements on the 40 most commonly used strains has provided a much needed platform upon which more complex phenotypes can be assessed [65]. Several large-scale studies have produced hundreds of models of human disease using chemical mutagenesis or random insertional mutagenesis [66]. Other projects are underway with the goal of producing a targeted mutation in every gene in the mouse genome [67]. As the primary organizational framework for all of these projects, it is essential that the annotated mouse genome reference sequence is as complete and accurate as possible.

### Sequencing Strategies

The original MGSCv3 mouse draft assembly proved comparatively cheap and easy to produce. A large number of other vertebrate genomes have been sequenced to similarly deep coverage, either as aids to model organism biology or to improve our understanding of the human genome. The cost to take a genome to an equivalent finished state is typically at least four times the cost of generating the draft assemblies using traditional Sanger sequencing. Nonetheless, it is clear from our analysis of the finished mouse genome assembly that draft WGSAs will always poorly reflect lineage-specific biology. This conclusion is also supported by analysis of both the dog [14],[68] and rhesus macaque genomes [16],[28]. Whereas they provide a sufficient framework for understanding the majority of the genome that lies outside of lineage-specific regions, recent, rapidly evolving parts of the genome are greatly underrepresented due to the collapse of segmental duplications and evolutionarily young repeat sequences. Finished genome sequence has proved essential to understanding the full range of biology for both the human [17] and the mouse genome, and will no doubt prove similarly informative for other vertebrate species.

Using next-generation sequencing technology, the cost of generating several-fold coverage of a genome drops several orders of magnitude; however, especially for large genomes; it is still not possible to generate a de novo assembly from the collection of such reads. While it is likely that de novo assembly of large genomes using next generation sequencing technologies will be achieved relatively soon, it is unlikely that these assemblies will represent these complex, lineage-specific regions any better than WGSAs generated using traditional Sanger technology. We have seen little evidence from next-generation sequence assemblies of genome or clones that segmental duplications can be adequately resolved with methods other than capillary sequencing of clones. For example, we recently completed an analysis with 96 clones, which contained structural variants and segmental duplications and, not surprisingly, those regions that remained unresolved (by 454 sequence data) were enriched in segmental duplications and large common repeats (Eichler EE, Kidd JM, Fulton RS, Chen L, Graves T, et al. unpublished data). Cost-effectiveness should not be the primary consideration for these regions. Studies of human disease and phenotypes in other organisms show conclusively that the content, copy, and structure are important. Short-read, next-generation sequencing technology, while a significant advance, will not comprehensively capture all of this complex sequence structure. Obtaining large insert clones for these regions is the key, but we need third-generation technology with longer-read lengths to assemble these complex regions accurately. Long-read technology developments [69] hold promise in this regard. A targeted clone-based approach to sequencing may thus be especially well justified for representative species on the lineage leading to human, comprising our closest primate lineages as well as other simians. This is because many of the critical changes that underlie biological innovations in primate lineages are likely to reside in precisely the rapidly evolving, segmentally duplicated regions that have proved so illuminating for the mouse. Coding and noncoding gene gains and losses in each lineage, and changes to their regulatory sequence, may allow us to elucidate the pressures that have shaped different regions of the human genome over recent evolutionary history. Changes in the repertoire of immune genes, for example, would help to explain the contrasting susceptibilities of primates to different pathogens and auto-immune diseases [70], while much of human reproductive biology and placental development, so distinct from those of rodents, may be understood in the context of changes in reproductive strategy among primates [71]. Other laboratory models, such as the rat, or species that lie at key branch points in the evolutionary tree leading to humans, or representatives of important lineages outside the eutherian mammals, such as marsupials, birds, and fish, may be similarly justified in having a finished genome sequence.

### Comprehensive Gene Lists

The greatest improvements to the mouse assembly have been to regions that are replete with rodent lineage-specific duplicated sequence. Segmental duplications that were previously found at negligible levels now constitute almost 5% of the genome. Many of these duplications harbour multiple rodent-specific genes that show a strong bias towards reproductive function. This suggests a role for either adaptive forces or clonal selection in shaping the mouse genome. The availability of these mouse genes now allows their experimental investigation.

The comparison of two finished mammalian genomes has enabled the revision of comprehensive and reliable human and mouse protein-coding gene catalogues. The 75% of mouse genes that are in 1∶1 orthologous relationships with human genes are the most likely to have maintained ancestral function in both species, and are, therefore, most appropriately targeted as disease models. Phenotype data, mainly from knockouts, are already available for over 5,000 of these 15,187 genes [72]. Other genes exist in multiple copies in the B6 genome or are polymorphic among mouse strains, and these will be more difficult to study on a gene-by-gene basis. While variation among strains suggests that some of these lineage-specific genes are not essential to development, many of these differences may contribute to phenotypic variation seen among laboratory strains. Understanding rodent-specific innovations is, therefore, critical when investigating human physiology or disease using the mouse as a model organism.

The shortcomings of the initial draft assembly are readily apparent now that a more-complete genome assembly is available. Undoubtedly these have led to incomplete or inaccurate understanding of some aspects of mouse biology. The availability of high quality genome sequence for the mouse will lead the way in dismissing some commonly held misconceptions and, more importantly, in revealing many previously hidden secrets of mouse biology.

Supplemental material and data for this paper including validated protein-coding and noncoding gene models can be found at: http://www.ncbi.nlm.nih.gov/genome/guide/mouse/Build36_Publication_Supplement).

## Materials and Methods

### Sequence Generation and Assembly Production

#### Clone-based sequence production

Ninety-six percent of the clone-based sequence was derived from four centres, The Genome Center at Washington University in St. Louis, The Wellcome Trust Sanger Institute, The Broad Institute of Harvard and MIT, and The Genome Center at the Baylor College of Medicine (Figure S6 in Protocol S1). DNA for the BAC, fosmid, and Whole Genome libraries was obtained from The Jackson Laboratories. Information on BAC library construction (libraries RP23, RP24, CH25, and CH36) can be found at BacPac Resources [73]. Clone sequences were obtained as previously described [19] with two exceptions. The availability of B6 WGS reads allowed the centres to sequence clones to a moderate level of coverage and then “steal” reads to increase the coverage. Remaining gaps were then finished as described previously [19]. Additionally, the requirements for finishing simple sequence repeats (SSRs) in full were changed to account for the increased levels of SSRs in the mouse genome. Additional information concerning centre-specific protocols for clone assembly processes can be found in the Protocol S1: Assembly Production.

#### Clone sequence quality assurance

To ensure that base level quality of the assembled clones was high, we performed a quality assurance exercise. Each sequencing centre provided the assessing centre with the clone-based shotgun traces they had produced. The assessing centre then used their internal protocols to steal reads and assemble the final insert sequences. The two sequences were then aligned, and all differences were manually assessed by an independent third party. Differences found within SSRs were not counted as true differences. The overall base level error rate was determined to be 1 error per 50,000 bp, well below the accepted finishing standard of 1 error in 10,000 bp (Table S6 in Protocol S1).

#### Tiling path production

The genome assembly is driven by a tiling path file (TPF). This provides information concerning clone (component) order as well as the location and characterization of gaps. Two methods were used to obtain clone order: alignment of clone end sequences to the MGSCv3 and clone order as obtained by the mouse fingerprint map [74]. All TPFs were stored and evaluated in a single system. Alignments between adjacent clones were produced using a script utilizing a combination of BLAST and a banded Needleman-Wunsch algorithm (Cherry J, unpublished data; Protocol S1: Tiling Path Production). Alignments having greater than 99.6% identity, no gap greater than 25 bp, and a complete dovetail alignment were “passed”. While it is expected that all clones should have an alignment of 100% identity with no gaps, in practice this rarely happens due to the difficulty of finishing SSRs. Clones not passing the above criteria were manually assessed. In some cases, manual adjustment of the alignment could produce an acceptable overlap and in other cases, a “certificate” was submitted to provide additional external evidence that the join was acceptable. For more information on join quality information Table S7 in Protocol S1.

#### Production of Build 36

Using the alignments above, AGP files were generated using a program called contig_build (Cherry J, unpublished data). This algorithm takes a tiling path and a set of alignments and generates a contig sequence. It checks for internal consistency with respect to clone order on the TPF and the provided alignments. The generated switch points were selected based on the component overlaps. In a few cases, switch points were manually edited to exclude contaminant sequence or misassembled sequence in one of the components.

To ensure inclusion of as much sequence as possible, the above assembled contigs were compared to the MGSCv3 and a combined assembly was generated essentially as previously described [75]. In this case, the MGSCv3 was used as a TPF for reconciliation of alignment conflicts. All regions where WGS sequence was incorporated were manually assessed and the most conservative path that minimized gaps was chosen. While the vast majority of sequence in Build 36 is finished (HTGS phase 3 sequence), there was a small amount of draft and WGS sequence included (Table S3a and S3b in Protocol S1). Inclusion of this sequence was necessary as some regions of the genome were recalcitrant to propagation in BAC vectors (Figure S9 in Protocol S1).

### Genome Analysis

#### Production of conserved synteny map

This was produced essentially as described previously [11].

#### Repeat analysis

Both Build 36 and the MGSCv3 were analyzed using RepeatMasker version open-3.1.3 with the following parameters: -w –s –no_is –cutoff 255 –frag 20000 –gff –species mouse [76].

#### Segmental duplication analysis

This was produced as described previously [5].

#### Variation

Mouse sequence reads were obtained from the NCBI Trace Archive; quality clipped, and repeat masked using WindowMasker [77]. Reads were then aligned to either assemble using BLAST (version 2.2.18) (-W 28 –e 0.0001 –m8 –UT –Fm –RT –nT) [78]. Only the highest scoring alignments were retained and the highest scoring alignment had to be at least 10% larger than the second-highest alignment score. After the top scoring alignment region had been identified, the region was padded by 20 kb on either side and the read was realigned using cross-match (P. Green, unpublished data). For an alignment difference to be scored as a variation, we required a unique alignment in the genome and confirmation by more than one trace that had been sequenced in a different sequencing tray. Additional information can be found in Protocol S1: Copy Number Variation.

In order to identify variation based on mate pair violations, the BLAST alignments described above were sorted by best hit. The top scoring hits for either end that were within 200 kb of each other were retained for further analysis. If multiple locations for a clone could be identified, the clone was not kept for the final analysis. We defined a placed read pair as “satisfied” if the calculated insert size was within three standard deviations of the mean. Additional information can be found in Protocol S1: Copy Number Variation.

#### Gene catalogue construction

Mouse and human gene models identified using either the Ensembl pipeline (release 43) or the NCBI pipeline (mouse Build36 v1 and human Build 36 v3) were obtained. Comparison of genomic coordinates allowed for the reconciliation of these two sets into a single gene catalogue (Protocol S1: Protein-Coding Genes and Gene Families).

The reconciled gene lists were quality assessed based on their predicted orthologous relationships as previously described [32] and on the conservation of exonic boundaries in the case of multi-exonic genes.

To determine genes that are missing from the MGSCv3, the Build 36 and the MGSCv3 assemblies were aligned to each other using BLAST [78]. The alignments were trimmed to maximize alignment scores and to retain reciprocal best hits. An additional step allowed alignments that are of high quality, but not reciprocal, to be included in order to capture regions that are duplicated in one assembly, but not in the other (Y. Kapustin, unpublished data). CDS features on Build 36 were then propagated through the alignments onto the MGSv3 (J. Cherry, unpublished data) to obtain coordinates on the MGSCv3. Exonic coordinates from the union of NCBI and Ensembl transcripts for each gene were mapped through alignments from Build 36 onto MGSCv3 (J. Cherry, unpublished data). Exonic regions that abutted, or were wholly contained within, one another were merged before mapping, as were MGSCv3 aligned regions that overlapped.

We have chosen not to compare the current and initial draft mouse gene catalogues, because gene annotations have benefitted from the many improvements in the availability of transcriptional evidence, gene prediction algorithms, and the heuristics used to evaluate these data. Instead, we determined the extent to which the initial MGSCv3 assembly could have supported the current mouse gene catalogue. We were thus interested in identifying disrupted Build 36 gene models whose corresponding MGSCv3 sequence was (i) not previously placed on chromosome scaffolds; (ii) previously dispersed among two or more different chromosomes, and/or were placed on both strands of a single chromosome; (iii) interdigitated, on the same strand, with sequence corresponding with an unrelated gene model; and (iv) entirely absent from this early assembly. We describe any such gene model as being “unmatched” in MGSCv3. These four unmatched criteria were applied in order. The remaining “matched” Build 36 gene models are contiguous and their exons do not overlap with other gene models on the same strand in both Build 36 and MGSCv3 assemblies. With few (65) exceptions, these gene models are placed on the same chromosome and strand in both assemblies.

For each Build 36 gene model, we then tabulated its exonic regions according to these four unmatched criteria. This allowed us to estimate the proportion of Build 36 exonic nucleotides that could have been predicted correctly in the early MGSCv3 assembly. Build 36 gene models were deemed to be “substantially disrupted” (see Main Text: Mouse and Human Protein Coding Gene Repetoires) in MGSCv3 if greater than 25% of its exonic sequence falls into any of these four categories.

Links to our gene catalogues and further details of these analyses can be found in Protocol S1 (Assembly Comparison and Analysis: Protein Coding Genes and Gene Families).

#### Identifying ancestral and derived ncRNAs

We looked for evidence of human transcription for a set of known, mouse long ncRNAs [63] using human ESTs and RNA sequences from GenBank. The coordinates of all human ESTs and RNAs available from GenBank that mapped uniquely to regions outside of known protein-coding genes in the human genome (Ensembl v50; 384,861 sequences) were mapped onto the mouse Build 36 assembly using the human–mouse genome alignment data and the LiftOver tool from University of California Santa Cruz [79]. We used default parameters and set the minimum ratio of mapped nucleotides to 0.2, which was appropriate for the human–mouse divergence. Of 145,321 sequences that map to the mouse assembly, only those found outside of known protein-coding genes in the mouse genome were considered for further analysis (96,367 human ESTs or RNAs). To identify candidate ancestral noncoding transcripts, we selected mouse ncRNAs from the previously described set of 3,051 mouse long noncoding genes that overlapped by one or more nucleotides these mapped human intergenic ESTs and RNAs. The statistical significance of this overlap was determined using a genome-wide association procedure that accounts for G+C-content and chromosomal biases [63]. The statistical significance for the overlap between mouse non-coding RNAs and the annotated mouse repeat-derived sequence [80] were determined similarly, using mouse intergenic sequence as the null model. The significance of the increased fraction of overlapping sequence between repetitive elements and conserved over nonconserved mouse ncRNAs was determined using a Fisher's exact test.

## Supporting Information

Protocol S1 Supporting figures, tables, and text. All supporting information can be found at the following Website: http://www.ncbi.nlm.nih.gov/projects/genome/guide/mouse/Build36_Publication_Supplement/.
(0.04 MB DOC)

## Abbreviations

EST: expressed sequence tag
ncRNA: noncoding RNA
SSR: simple sequence repeat
TPF: tiling path file
VR: vomeronasal receptors
WGSA: Whole Genome Sequence and Assembly
