# Supplementary material for: Lineage-Specific Biology Revealed by a Finished Genome Assembly of the Mouse
Source: PLoS Biol. 2009 May 26;7(5):e1000112. doi: 10.1371/journal.pbio.1000112 (PMC2680341; doi:10.1371/journal.pbio.1000112)
Supplement: Protocol S1 — Supporting figures, tables, and text. All supporting information can be found at the following Website: http://www.ncbi.nlm.nih.gov/projects/genome/guide/mouse/Build36_Publication_Supplement/. (0.04 MB DOC) [file pbio.1000112.s001.doc]

[**http://www.ncbi.nlm.nih.gov/projects/genome/guide/mouse/Build36_Publication_Supplement/**](http://www.ncbi.nlm.nih.gov/projects/genome/guide/mouse/Build36_Publication_Supplement/)

These pages provide supplemental data supporting information contained in the [PLoS Biology](http://biology.plosjournals.org/perlserv/?request=index-html&issn=1545-7885) publication describing the mouse genome (ref). There is information concerning assembly metrics for the initial draft assembly (MGSCv3), Build 36 (analyzed in the publication) and Build 37 (released on July 10, 2007).

## Table of Contents

### [Assembly Production](http://www.ncbi.nlm.nih.gov/projects/genome/guide/mouse/Build36_Publication_Supplement/AssemblyProduction.html)

- [Bases by Center:](http://www.ncbi.nlm.nih.gov/projects/genome/guide/mouse/Build36_Publication_Supplement/AssemblyMetrics.html#CENTER) Non-redundant contribution of bases produced by each center that were used in Build 36.
- [Clone Sequence Production:](http://www.ncbi.nlm.nih.gov/projects/genome/guide/mouse/Build36_Publication_Supplement/AssemblyProduction.html#CloneSequence)A description of sequencing centers use of WGS reads in BAC sequence production.
- [Clone QA:](http://www.ncbi.nlm.nih.gov/projects/genome/guide/mouse/Build36_Publication_Supplement/AssemblyProduction.html#QA) Quality assessment of clones to ensure differing methods of WGS read incorporation did not affect the quality of the finished clones.
- [Tiling Path Production:](http://www.ncbi.nlm.nih.gov/projects/genome/guide/mouse/Build36_Publication_Supplement/AssemblyProduction.html#JOIN) Generation of tiling path and curation of finished clone overlaps.
- [Build 36 Details:](http://www.ncbi.nlm.nih.gov/projects/genome/guide/mouse/Build36_Publication_Supplement/AssemblyProduction.html#BUILD36) More details concerning the generation of Build 36.

### [Assembly Metrics](http://www.ncbi.nlm.nih.gov/projects/genome/guide/mouse/Build36_Publication_Supplement/AssemblyMetrics.html)

- [Chromosome Lengths:](http://www.ncbi.nlm.nih.gov/projects/genome/guide/mouse/Build36_Publication_Supplement/AssemblyMetrics.html#LENGTH) Calculation of chromosome length, including estimated gaps.
- [Number of bases (without Ns):](http://www.ncbi.nlm.nih.gov/projects/genome/guide/mouse/Build36_Publication_Supplement/AssemblyMetrics.html#BASES) Calculation of chromosome lengths, excluding estimated gaps.
- [Base Composition:](http://www.ncbi.nlm.nih.gov/projects/genome/guide/mouse/Build36_Publication_Supplement/AssemblyMetrics.html#COMPOSITION) Status of bases contributing to the assembly (finished, draft, WGS, gaps).
- [Continuity (N50):](http://www.ncbi.nlm.nih.gov/projects/genome/guide/mouse/Build36_Publication_Supplement/AssemblyMetrics.html#CONTINUITY) The length at which half of the bases in an assembly reside in a scaffold of at least that length.
- [Gap information:](http://www.ncbi.nlm.nih.gov/projects/genome/guide/mouse/Build36_Publication_Supplement/AssemblyMetrics.html#GAP) The number of spanned and un-spanned gaps in the assembly.

### [Assembly QA](http://www.ncbi.nlm.nih.gov/projects/genome/guide/mouse/Build36_Publication_Supplement/AssemblyQA.html)

- [Alignments of finished clones to MGSCv3](http://www.ncbi.nlm.nih.gov/projects/genome/guide/mouse/Build36_Publication_Supplement/AssemblyQA.html#PH3_MGSCv3):Alignemnt of finished clones to the MGSCv3.s
- [Assembly Alignments:](http://www.ncbi.nlm.nih.gov/projects/genome/guide/mouse/Build36_Publication_Supplement/AssemblyQA.html#Alignments)Build 36 and MGSCv3 were directly aligned using megaBLAST to assess coverage and assembly differences.
- [STS content and comparison to non-sequence based maps:](http://www.ncbi.nlm.nih.gov/projects/genome/guide/mouse/Build36_Publication_Supplement/AssemblyQA.html#STS)STSs used in the construction of two non-sequence based maps are used to anchor these maps to the assembly and look for differences.
- [RefSeq Alignments:](http://www.ncbi.nlm.nih.gov/projects/genome/guide/mouse/Build36_Publication_Supplement/AssemblyQA.html#REFSEQ) RefSeq transcripts are aligned to Build 36 in order to assess Build coverage.
- [C57BL/6J Paired end read alignments:](http://www.ncbi.nlm.nih.gov/projects/genome/guide/mouse/Build36_Publication_Supplement/AssemblyQA.html#PairedEnds)End sequences from clones derived from C57BL/6J were aligned to Build 36 and consistency checks were performed to assess Build 36.
- [Comparison to the optical map:](http://www.ncbi.nlm.nih.gov/projects/genome/guide/mouse/Build36_Publication_Supplement/AssemblyQA.html#OpticalMap)Build 36 was compared to an optical map derived from Build 36 to assess clone integrity and assembly consistency.

### [Assembly Analysis and Comparison to MGSCv3](http://www.ncbi.nlm.nih.gov/projects/genome/guide/mouse/Build36_Publication_Supplement/AssemblyComparison.html)

- [Repeat Data:](http://www.ncbi.nlm.nih.gov/projects/genome/guide/mouse/Build36_Publication_Supplement/AssemblyComparison.html#REPEAT)Informtion on Repeat Masker analysis
- [Protein-Coding Genes and Gene Families:](http://www.ncbi.nlm.nih.gov/projects/genome/guide/mouse/Build36_Publication_Supplement/AssemblyComparison.html#GENES) Reconciliation and evaluation of gene models. Also includes comparison of gene models to the human gene set.
- [Non-coding Transcripts:](http://www.ncbi.nlm.nih.gov/projects/genome/guide/mouse/Build36_Publication_Supplement/AssemblyComparison.html#NON_CODING)Information on mouse non-coding transcripts with human orthologs
- [Copy Number Variation:](http://www.ncbi.nlm.nih.gov/projects/genome/guide/mouse/Build36_Publication_Supplement/AssemblyComparison.html#CNV)Sequences from non-C57BL/6J strains were aligned to Build 36 and the MGSCv3 to identify copy number variation.
